# PhenoCams for Field Phenotyping: Using Very High Temporal Resolution Digital Repeated Photography to Investigate Interactions of Growth, Phenology, and Harvest Traits

**DOI:** 10.3389/fpls.2020.00593

**Published:** 2020-06-18

**Authors:** Helge Aasen, Norbert Kirchgessner, Achim Walter, Frank Liebisch

**Affiliations:** ^1^Group of Crop Science, Department of Environmental Systems Science, Institute of Agricultural Sciences, ETH Zürich, Zurich, Switzerland; ^2^Water Protection and Substance Flows, Research Division Agroecology and Environment, Agroscope, Zurich, Switzerland

**Keywords:** high-resolution remote sensing, crop phenology, leaf area index, early vigor, seasonal crop development, dynamic traits, soybean, agriculture

## Abstract

Understanding the interaction of plant growth with environmental conditions is crucial to increase the resilience of current cropping systems to a changing climate. Here, we investigate PhenoCams as a high-throughput approach for field phenotyping experiments to assess growth dynamics of many different genotypes simultaneously in high temporal (daily) resolution. First, we develop a method that extracts a daily phenological signal that is normalized for the different viewing geometries of the pixels within the images. Second, we investigate the extraction of the in season traits of early vigor, leaf area index (LAI), and senescence dynamic from images of a soybean (Glycine max) field phenotyping experiment and show that it is possible to rate early vigor, senescence dynamics, and track the LAI development between LAI 1 and 4.5. Third, we identify the start of green up, green peak, senescence peak, and end of senescence in the phenological signal. Fourth, we extract the timing of these points and show how this information can be used to assess the impact of phenology on harvest traits (yield, thousand kernel weight, and oil content). The results demonstrate that PhenoCams can track growth dynamics and fill the gap of high temporal monitoring in field phenotyping experiments.

## Introduction

Understanding the interaction of growth dynamics and phenology with environmental conditions is crucial to improve the resilience of our cropping systems to a changing climate (Pretty et al., 2010; Asseng et al., 2013, 2015; Siebert and Ewert, 2014). One approach to this is to characterize the growth of many different genotypes in different environments. Phenomics or phenotyping aims to describe the appearance of plants as a function of the interaction of its genetic background with environmental conditions and is currently one of the most rapidly developing disciplines in crop science (Fiorani and Schurr, 2013; Walter et al., 2015). New platforms and imaging techniques (Li et al., 2014; Cendrero-Mateo et al., 2017; Hund et al., 2019) aim to facilitate high-throughput analyses of plant traits. For field phenotyping applications, where plants need to be monitored in outdoor environments, the development of standardized methods for plant trait mapping is crucially needed (White et al., 2012; Busemeyer et al., 2013; Deery et al., 2014; Kirchgessner et al., 2017; Virlet et al., 2017). This setting, referred to as field phenotyping, has been identified as one of the largest challenges in plant phenotyping (Pieruschka, 2016; Araus et al., 2018).

Today, the most common technique to assess the growth of many different varieties under field conditions is still the visual rating by trained experts. In recent years, large infrastructures have been put into practice that can screen crop traits in a (semi-) automatic fashion (Kirchgessner et al., 2017; Virlet et al., 2017). Also unmanned aerial systems (Bendig et al., 2015; Liebisch et al., 2015; Zaman-Allah et al., 2015; Gómez-Candón et al., 2016; Haghighattalab et al., 2016; Kefauver et al., 2017; Madec et al., 2017; Yang et al., 2017; Aasen and Bolten, 2018; Aasen et al., 2018; Hu et al., 2018; Roth et al., 2018) and manual and (semi-) automated ground-based approaches (Gnyp et al., 2013; Tilly et al., 2015; Madec et al., 2017; Jimenez-Berni et al., 2018) are now used for this purpose. All of these approaches can deliver very high spatial resolution data. However, dynamic processes such as phenology might only differ by a couple of days between different genotypes of the same crop species or under different environmental conditions (e.g., Anderegg et al., 2020). Thus, methods that can deliver data at very high temporal resolution (at a daily rate) of many experimental plots at the same time are needed in the context of plant breeding and variety testing.

In the field of ecology, the technique of digital repeat photography—also called PhenoCams—that continuously capture images of a given area with an RGB or near-infrared-enabled cameras has been used for more than a decade to estimate phenology (Richardson et al., 2007, 2009, 2018a; Ahrends et al., 2009; Penuelas et al., 2009; Graham et al., 2010; Ide and Oguma, 2010; Kurc and Benton, 2010; Migliavacca et al., 2011; Keenan and Richardson, 2015; Hufkens et al., 2016). Usually, the cameras are mounted on towers or lookouts and view the canopy horizontally or obliquely to record the objects within their field of view several times a day. Within these images, the brightness information of the RGB channels can then be used to track changes in phenology. Based on the digital numbers (DN) of the RGB channels, different color indices (CIs) such as the green or red chromatic coordinates or the excess green index were evaluated for that purpose. These CIs normalize the scene illumination or have an improved capability to distinguish vegetation (Sonnentag et al., 2012). Based on CIs derived with Phenocams, many studies have investigated the timing of phenological events [also called phenological transition dates (Richardson, 2019)], such as budburst and leaf-out (Klosterman et al., 2014) or the start and end of the season, as well as the peak of redness and end of redness during senescence (Xie et al., 2018). Different software packages have been published to process PhenoCam data. PHENOR in combination with the PHENOCAM and DAMETR R package allows extraction and modeling of phenological information from images. Hufkens et al. (2018a) showed how this data could be used to evaluate phenology models. The Phenopix R package automates different fitting techniques as well as the extraction of phenological events (Filippa et al., 2016). Since different terminologies are used throughout the literature, we will use the term “PhenoTimePoint” as a point in time that refers to an event within the phenological signal and the term “PhenoPhase” as a temporal period during the phenological development.

Most studies have been carried out on natural or semi-natural vegetation and rarely on intensively managed agricultural fields and never to distinguish crop genotypes or varieties. Anderson et al. (2016) and Andresen et al. (2018) investigated the growth of vegetation in high arctic regions and Snyder et al. (2016) the PhenoPhases in a cold desert. Several studies have investigated grassland (Migliavacca et al., 2011; Julitta et al., 2014; Hufkens et al., 2016; Browning et al., 2017; Liu et al., 2017; Fan et al., 2018; Filippa et al., 2018) and forest phenology (Richardson et al., 2007, 2013, 2018b; Hufkens et al., 2012; Sonnentag et al., 2012; Keenan et al., 2014; Klosterman et al., 2014; Reid et al., 2016; Donnelly et al., 2017; Liu et al., 2017, 2018; Filippa et al., 2018; Toda and Richardson, 2018). Throughout the last years, large datasets of PhenoCam imagery that cover several different vegetation types have been established (Brown et al., 2016; Hufkens et al., 2018b; Nagai et al., 2018; Richardson et al., 2018a).

Still, not too many studies have been published for agricultural crops. Sakamoto et al. (2012) used two compact digital cameras to capture visible and near-infrared images to observe seasonal changes in crop growth of maize and soybean (*Glycine max*) during one year. They found that the camera-derived CIs were closely related to VIs calculated using a multispectral sensor (SKYE) and MODIS satellite reflectance. Additionally, they found that CIs were related to the change of green LAI, total LAI, and above-ground dry biomass of stalks and leaves during the season. Zhu et al. (2016) used a tower-based system to detect the wheat heading stage based on ear feature detection within the images and Brocks and Bareth (2018) used stereo images from a tower based system to estimate biomass in barley.

To the authors’ knowledge, the PhenoCam approach has not yet been used (i) in the typical field phenotyping setting, where the growth of multiple genotypes needs to be characterized simultaneously, and (ii) to investigate the impact of the timing and duration of PhenoPhases on harvest parameters. The aim of this paper is to investigate the PhenoCam approach within a field phenotyping setting, in which the performance of several genotypes is compared. First, we will introduce a data processing workflow that takes into account the special needs of crop monitoring in the context of field phenotyping. Then, we will follow the phenological signal throughout a season and evaluate the data for a soybean variety testing trial. We show how the phenological signal can be used to estimate in season traits such as early vigor, dynamic traits such as LAI development and senescence dynamics, which are very important traits in the context of plant breeding (Hund et al., 2019). Additionally, we demonstrate how information on phenological timing can be used to assess the impact of phenology on harvest traits such as thousand kernel weight (TKW), yield, and oil content.

## Methodology

**FIGURE 1:**
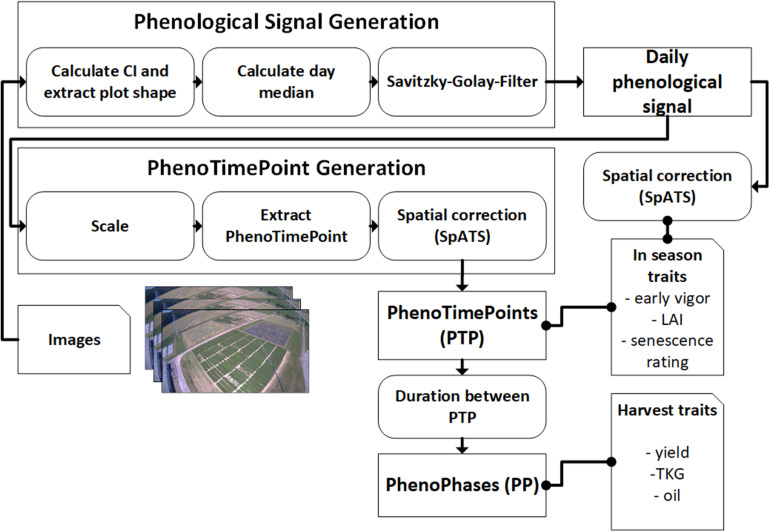
Schematics for the methodology of using PhenoCams for field phenotyping. Arrows point in the direction of data flow. Dotted connections represent data correlation. Both the in-season and harvest traits are also spatially corrected for in-field heterogeneity.

In the following, the dataset and methodology to extract in season and harvest traits from the phenological signal are introduced. Figure 1 gives an overview of the workflow.

### Camera System and Dataset Description

**FIGURE 2:**
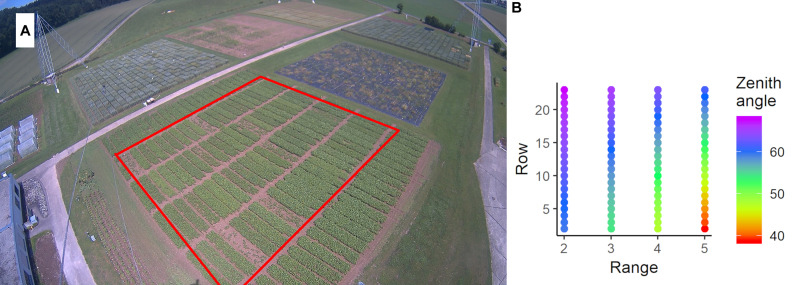
**(A)** Image captured by the FIP-PhenoCam system on the June 19, 2015. The red rectangle denotes the relevant plots of the soybean experiment. The experiment is surrounded by one row of border plots. At the top-left of the image, the wheat experiment is visible. In the foreground, two cables of the field phenotyping platform are visible. **(B)** Average zenith viewing angle for each plot.

We use images of a LUPUSNET HD – LE971 camera^1^, which provides a resolution of 1,920 × 1,080 pixels. It was mounted on a pole of the ETHZ field phenotyping platform (Kirchgessner et al., 2017) at 24.5 m height above the corner of our research field at the ETH Research Station for Plant Sciences Lindau-Eschikon, Switzerland (47.449 N, 8.682 E; 556 m above sea level). Viewing direction was toward north, north-west. In 2015, we used 44,000 images recorded with a frequency of 4 to 60 images per hour. Figure 2 shows an example image of the soybean field from 2015.

The soil of the experimental field was slightly acidic (pH = 6.2) cambisol with sandy loam soil structure and low humus content. The soil analysis indicated sufficient availability for phosphate, potassium, and magnesium. 10 soybean cultivars (*Glycine max* [L.] Merrill) differing in the time needed for seed maturation and in nutritional food composition of the beans were selected (Table 1). All of the cultivars were commercially available in Switzerland, and eight of them were on the national list of Swiss soybean varieties (Schwärzel and Hiltbrunner, 2015). 88 experimental plots of 1.5 m by 6.5 m containing seven rows of a particular soybean cultivar were sown on April 10, 2015. Each cultivar was planted in eight replications in a randomized complete block design, whereas four were used for ground validation measurements. During the season between the sowing date (10.04.2015) and the harvest (10.09.2015), crop cultivation measures were done according to best management practices for breeding trials. The genotype “Amphor” was excluded from analysis due to low viability resulting from old seed age (plots with bad growth in Figure 2A). The details on the set of genotypes are given in Table 1 (see Supplementary Table S2 for details of field management).

**TABLE 1:** Evaluation of oil and protein contents as well as the yield of the investigated soybean cultivars and the classification into the different maturity groups: +++, very good; ++, good; +, intermediate to good; Ø, intermediate; –, weak to intermediate; NA, not available [according to (Schwärzel and Hiltbrunner, 2015)].

Cultivar	Maturity group	Difference in growth days^1^	Oil content	Protein content	Yield^3^
Aveline	Medium early	−4	–	++	+
Falballa	Medium late	NA	–^2^	+++^2^	–(Ø)^2^
Gallec	Early	−6	–	+	++
Lissabon	Medium early	−2	–	+	++
Merlin	Early	−8	+	+	++
Obelix	Early	−5	+^2^	Ø^2^	+++
Opaline	Medium late	1	+	+	++
Proteix	Medium late	1	+	++(+)	++
Tiguan	Very early	NA	+^2^	Ø^2^	++^2^
Turmaline	Medium late	NA	+^2^	+^2^	+++^2^

*The cultivars “Falbala” and “Tiguan” were not recommended according to the Swiss cultivars catalog (Schwärzel and Hiltbrunner, 2015). ^1^Reference cultivar Maple Arrow (0 days), ^2^www.dsp-delley.ch, 05.03.2020, ^3^Within the maturity group.*

### Phenological Signal Generation

#### Extraction of Plot Values

To extract a representative value for each plot, we created a plot mask with a margin sufficient to keep the mask inside the plot’s extent during the complete growing period. This is important, since the plot’s position in the image slightly changes during the growing period due to the interaction of viewing angle and crop height. These masks are used to extract the RGB values of the plots. Depending on the position in the image, the masks of each plot contain between 1,000 and 9,400 pixels. The green chromatic coordinate (gcc; Gillespie et al., 1987) allows estimating the “greenness” of objects (Hufkens et al., 2012; Sonnentag et al., 2012; Brown et al., 2016; Richardson, 2019). It divides the green pixel value by the sum of the pixel values of the green, red, and blue channels (Eq. 1). Previous studies showed that gcc compensates changing illumination conditions and can be used with non-calibrated cameras (e.g., Sonnentag et al., 2012). To exclude the influences of obstacles such as cables of the field phenotyping platform, we averaged all gcc values within the 25th to 75th percentiles of the pixels in each mask. We apply this methodology to all day images of the dataset. As a result, we get gcc values for each plot with a temporal resolution of the capturing frequency of the image.

(1) gcc=green/(green+red+blue)

#### Extraction of Daily Signal

**FIGURE 3:**
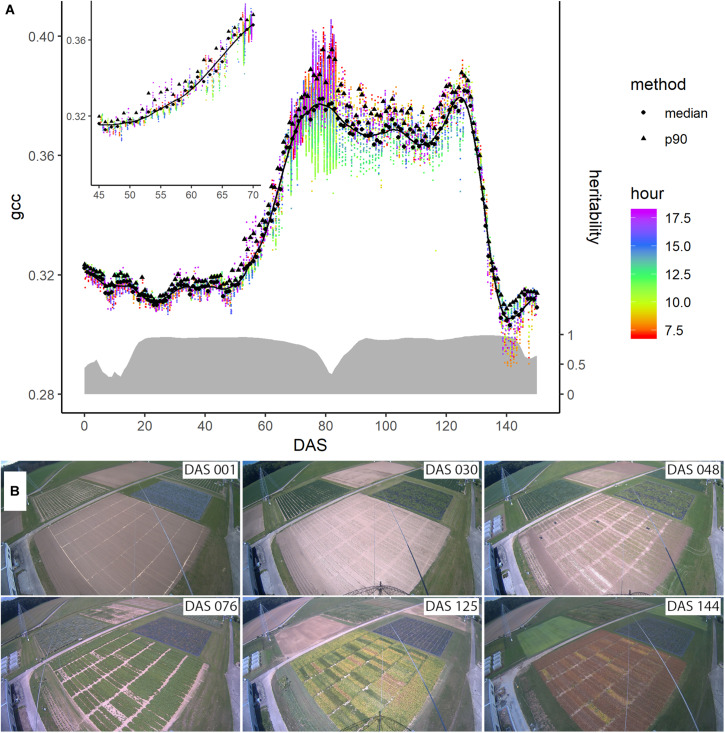
**(A)** Evolution of the phenological signal of plot 4 denoted by the green chromatic coordinate (gcc) during the season. The colored dots represent the individual data points extracted from the images, while their color corresponds to the measurement time (around DAS 80 the measurement frequency of the camera was increased for a short period). The black dots and triangles are the daily averages derived from the median and the 90th percentile, respectively. The solid line represents the Savitzky-Golay smoothed median values. On the right axis and denoted with the gray ribbon is the corresponding heritability of the signal across the genotypes measured by means of heritability (H^2^) across the season. **(B)** Images taken by the digital repeat photography device at six different days, denoted in days after sowing (DAS), during the growing season of soybean.

Different methods have been proposed to get a representative signal for each day. Often, the 90th percentile of all values per day, along with a 3-day moving average is used to minimize day-to-day variation mainly caused by changing weather conditions influencing the illumination (Hufkens et al., 2012; Sonnentag et al., 2012). Other studies used a sigmoid function or logistic functions to normalize for these effects (Klosterman et al., 2014). After trying the median, mean, and different percentiles, we use the median per day to get a representative value per day, since we found that it is robust toward illumination changes (c.f. Figure 3). Nevertheless, the data is not fully free of day-to-day noise, which complicates the following analysis steps. Thus, we applied a Savitzky-Golay filter (Savitzky and Golay, 1964) with a 3rd order polynomial and frame length of 7, which is used widely to investigate time series data (Chen et al., 2004; Jönsson and Eklundh, 2004; Cao et al., 2018). The result is a daily phenological signal for each plot.

### PhenoTimePoint and PhenoPhase Extraction

**FIGURE 4:**
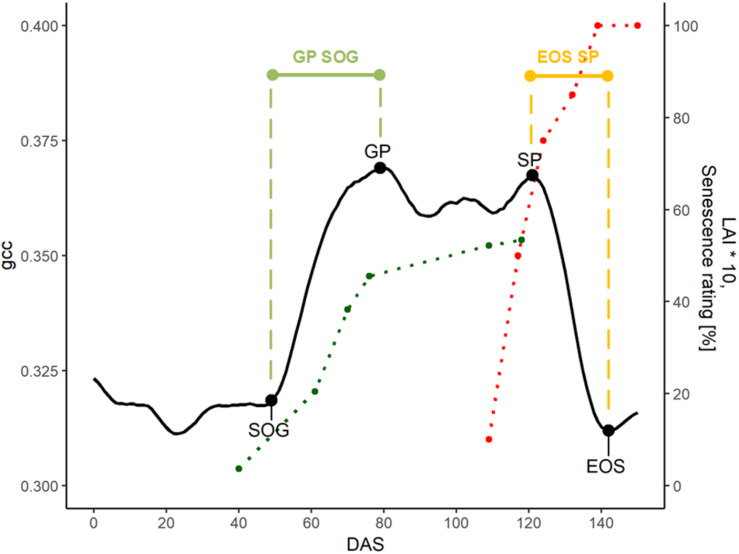
Overview of the phenological signal of plot 5 with different PhenoTimePoints: start of green up (SOG), green peak (GP), senescence peak (SP), and end of season (EOS). The light green and orange bars symbolize the PhenoPhases SOG to GP and SP to EOS, respectively. The red and green points correspond to the visual senescence rating and LAI development (multiplied by 10 for visualization purposes), respectively. The dotted lines are printed for visual reference.

To extract the PhenoTimePoints, we scaled the uncorrected phenological signal for each plot from 0 to 1. Then, we extracted significant points from the phenological signal by identifying minima and maxima and slope-based features. At the beginning of the green up phase, we defined the start of green up point (SOG) as the point in time at which the difference between two consecutive days increased above 0.015, which corresponds to a slope of 0.015, since the values were scaled between 0 and 1. The first maximum of the signal after the green-up was defined as green peak (GP). The last peak was defined as senescence peak (SP). The first minimum after the SP was defined as end of senescence/season (EOS). Table 2 summarizes the extracted features. For each plot, we extracted the days after sowing (DAS) for the identified PhenoTimePoint and corrected them for spatial effects (c.f. section “Spatial Correction”). Then, we calculated the duration of the PhenoPhases as the difference in DAS between two PhenoTimePoints. Figure 4 shows the concept for data of one plot.

**TABLE 2:** PhenoTimePoints with their abbreviation and description.

Abbreviation	Name of feature	Description
SOG	Start of green up	Rapid increase in greenness begins, slope >0.015 before green peak
GP	Green peak	Maximal gcc during green up
SP	Senescence peak	Maximum gcc during senescence
EOS	End of senescence (/Season)	First day with slope >−0.01 after SP

### Spatial Correction

Within breeding trials, it is common to correct for field heterogeneity resulting from differences in the soil or from slight differences in the management. Multiple approaches exist to correct for spatial effects (Gilmour et al., 1997; Piepho and Williams, 2010). Velazco et al. (2017) compared different mixed model approaches to correct for spatial trends. They found that the ‘Spatial Analysis of field Trials with Splines’ (SpATS) approach (Rodríguez-Álvarez et al., 2018), which uses two-dimensional P-splines, performed comparably to more elaborate and trial-specific spatial models, with the advantage of being flexible and user-friendly. It calculates the field heterogeneity into the following model:

(2) $$Y=f\left(r,c\right)+Z_{g}c_{g}+Z_{r}c_{r}+\varepsilon$$

where Y is the measured phenotypic value and *f*(*r*,*c*) is a smoothed bivariate surface defined over row (r) and range (c) positions across the field. The vector c_g_ is the random coefficient of the genotypes associated with the design matrix Z_*g*_ of the experiment, c_r_ is the random coefficient of the rows associated with design matrix Z_r_, and ε is the random error vector (Rodríguez-Álvarez et al., 2018).

Besides the field heterogeneity, our data is additionally influenced by the different viewing geometries within the image (c.f. Figure 2). Since the viewing geometry changes continuously across the image and consequently across the field, we consider it as a continuous component of spatial heterogeneity that adds to the spatial effects resulting from field heterogeneity and thus becomes part of the *f*(*r*,*c*) term.

With SpATS, we calculated the Best Linear Unbiased Estimator (BLUE) for each genotype; *Best*, meaning they have the lowest variance, *Linear*, meaning they are linear functions of the data, and *Unbiased*, means the expected value of a mean estimate for an individual equals its true value (Molenaar et al., 2018). While best linear unbiased predictions (BLUPs) have some advances compared to BLUEs in many cases (Piepho et al., 2008; Molenaar et al., 2018), in our case the limited number of genotypes and the lack of information on relationships to a base population did not allow to accurately estimate the genetic variance components. Thus, we set genotype effects as fixed and used BLUEs to normalize the field heterogeneity and compare between genotypes.

Heritability measures the proportion of the overall observed phenotypic variance that can be explained by the genetic variance. Heritability of the spatially corrected traits was calculated according to (Rodríguez-Álvarez et al., 2018) based on the genetically effective dimensions provided by SpATS with genotypes set as random effect:

(3) $$H_{s}^{2}=\frac{ED_{g}}{m_{g}-1}$$

where ED_g_ is the effective dimension for the genotypes and m_g_ is the total number of genotypes evaluated. The denominator (m_g_ – 1) reflects the upper bound for the effective dimension (see Rodríguez-Álvarez et al., 2018 for further details).

We calculated genotypic BLUEs and heritability for the phenotypic gcc values of every measurement date, the PhenoTimePoints before calculating the PhenoPhases and the field measured crop traits. Throughout the manuscript, the spatial corrected genotypic BLUEs are referred to as genotypic values, whereas the observed values are referred to as phenotypic values.

### In-Season Trait Estimation

To evaluate and validate the feasibility and relevance of the extracted PhenoTimePoints and PhenoPhases in relation to crop growth and development in a breeding trial context, we selected early vigor, leaf area index (LAI) and senescence. All these traits are known to be an important selection criteria for soybean breeding in Switzerland and similar environments.

Early vigor was rated as an indicator for plant health and growth development at 40 DAS. The plots were rated by two persons, independently of the germination rate, and the values were averaged. The used scale ranged from one to eight. One means that the plant is small and depicts a low vitality, whereas eight stays for very vigorous plants showing a large and vital plant habitus. The rating mainly integrates size and color information (Peter et al., 2009). The LAI was measured in regular intervals during homogeneous weather conditions such as cloud-free sky or constant cloud cover with an LAI-2200 Plant Canopy Analyzer (LI- COR, Inc., Lincoln, NE, United States). The LAI measurement combined a first measure above the canopy, followed by ten representative measurements diagonally spread through the plot canopy followed by a second above canopy measurement. With a 270° view-restricting cap, the potential negative influence by the measuring person was reduced. The senescence was estimated as the percentage of yellow and brownish colored leaves as well as fallen leaves according to (Munger et al., 1997). The senescence rating was done six times for each plot at 109, 117, 124, 132, 137, and 150 DAS, respectively. For each trait, we calculated the genotypic BLUEs and correlated both the (spatially not corrected) phenotypic and genotypic BLUEs with the uncorrected and spatially corrected phenological signal.

### Harvest Trait Estimation

To evaluate the relevance of the extracted PhenoTimePoints and PhenoPhases for overall crop performance, we investigated their relationship to the harvest traits yield, thousand-kernel weight (TKW), and seed oil content. Yield in t ha^–1^ was estimated using the seeds of all hand-harvested pods of 1 m of plants dried for 3 days at 40°C. Due to the experimental setup, machine harvest was only possible at the time of maturity of the latest ripening genotypes, when early genotypes were already affected by pod and kernel fall. The TKW (g) was calculated by counting and weighing the seeds of 10 randomly selected pods. Oil content (percentage of dry kernel weight) was measured in dry grains with a near-infrared spectrometer (NIRS, Infratec 1241 grain analyzer, Foss GmbH, Rellingen, Germany) using the company provided soybean program. Also, for the harvest traits, we calculated the genotypic BLUEs and correlated these with the BLUEs of the PhenoTimePoints and the duration of the PhenoPhases.

## Results

### Phenological Signal

Figure 3 shows the evolution of the gcc-based phenological signal of plot 4. After sowing, the phenological signal did not change much until around DAS 50. During this time, the vegetation growth was very limited due to the low temperatures. The fluctuations were mainly due to changes in soil moisture due to rain, although at DAS 23, the growth also started. At DAS 48, the gcc steeply started to increase. This increase was followed by a shoulder of high greenness around DAS 76, after which the greenness decreased before it rose again to a second peak around 125 DAS. After this second peak, the greenness steeply decreased until around DAS 144.

The black triangles and dots in Figure 3 represent two different approaches to extract a daily signal, namely the 90th percentile often found in literature and the median, respectively (c.f. subsection “Extraction of Daily Signal”). In direct comparison, the median values appeared less noisy than the 90th percentile, in particular when the environmental conditions differed, such as between DAS 50 and 60. Thus, we continued with the daily median to generate the phenological signal. Still, the median was also affected by changing illumination conditions, but the Savitzky-Golay filter was able to reduce these day-to-day fluctuations. The heritability of the phenological signal varied. Between DAS 15 and DAS 20, it increased to 0.95. At DAS 55, it slightly decreased to 0.925 at DAS 60, 0.85 at DAS 70, and 0.33 at DAS 80. Toward DAS 90, it increased again over 90 before it decreased again after DAS 140.

### Spatial Correction

**FIGURE 5:**
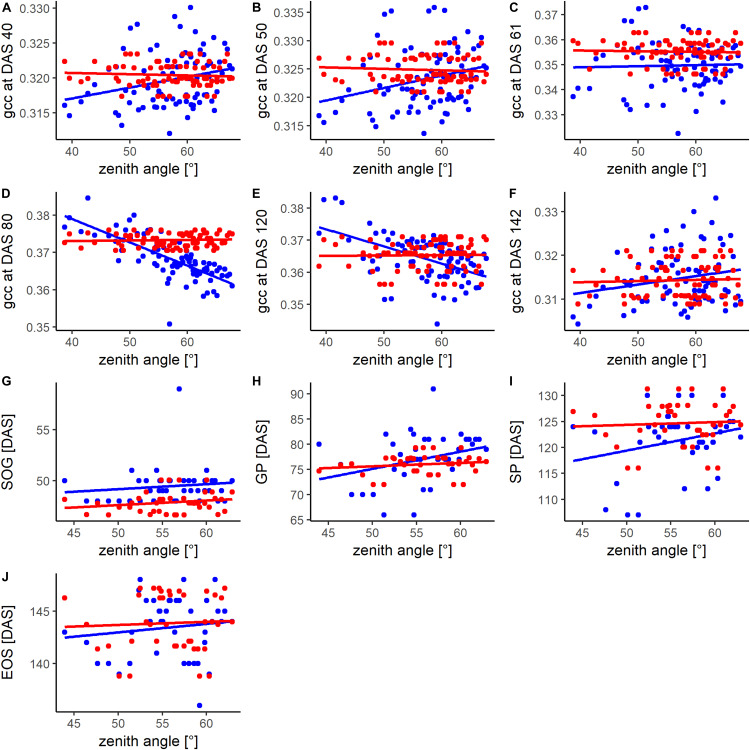
Comparison of the relationship between the zenith angle and the phenotypic (blue) and spatially corrected genotypic (red) values of the **(A–F)** phenological signal (gcc) at different days after sowing (DAS) and for different PhenoTimePoints **(G–J)**. For the DAS, information of all available plots (80) was used while for the PhenoTimePoints, only plots (40) where ground validation data was available were considered. For coefficient of determination and *p*-value please refer to Supplementary Table S3.

Figure 5 shows the phenotypic and genotypic values of the different plots of the phenological signal at different DAS and for different PhenoTimePoints in relation to the viewing geometry. The influence of the viewing geometry on the phenological signal changed throughout the season. During the early development (DAS 40 and 50), the signal was positively related to viewing geometry. During the green up (DAS 61), only a weak trend was found. Around the GP (DAS 80) and SP (DAS 120), the relationship was negative. At the end of the season, only a weak positive trend was observed. Overall, the viewing geometry had only a weak relationship with the signal (*R*^2^ < 0.08; Supplementary Table S3). For DAS 80 and 120, the viewing geometry had an *R*^2^ of 0.55 and 0.26 with the gcc (Supplementary Table S3). In all cases, the spatial correction could mitigate the viewing geometry effect. In all cases, the timing of the PhenoTimePoints had a weak positive trend with the viewing geometry (*R*^2^ < 0.09; Supplementary Table S3). Also here, the spatial correction could reduce this trend. The exact quantitative description of the relationships can be found in Supplementary Table S3.

**FIGURE 6:**
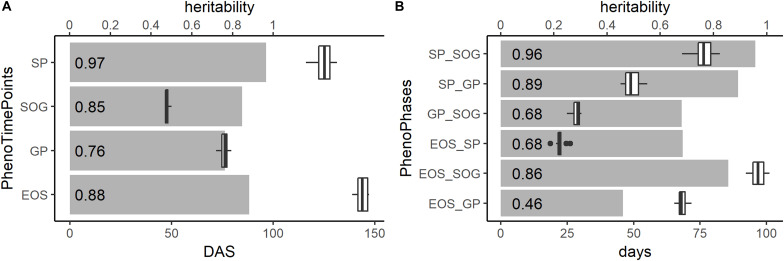
Heritability (H^2^; gray bars and number at the front of the bar), timings in days after sowing (DAS; boxplots) and duration in days of the PhenoTimePoints **(A)** and of the PhenoPhases **(B)**, respectively.

The gray bars in Figure 6 show the heritability of the PhenoTimePoints and PhenoPhases. After spatial correction, all PhenoTimePoints showed a high heritability (H^2^) above 0.76. Besides of the PhenoPhase EOS_GP (H^2^ of 0.46), all PhenoPhases also showed high to very high heritability (0.68–0.97). For exact timings with their variability and heritability, see Supplementary Table S1.

### In Season Trait Estimation of Leaf Area Index, Early Vigor, and Senescence

**FIGURE 7:**
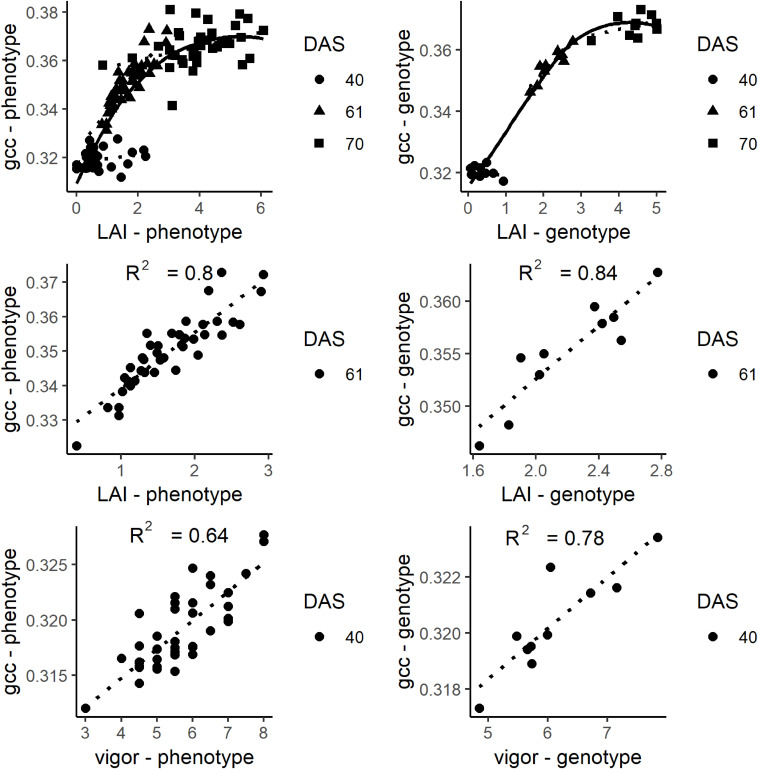
Top: Plots of gcc and leaf area index (LAI) at days after sowing (DAS) 40, 61, and 70. Middle: Plot of the relationship of LAI and gcc at DAS 61. The solid line is a smoothed line though all data points while the dotted lines are regression lines for each DAS. Bottom: Plots of gcc and vigor rating. On the left, the phenotypic values are shown. On the right, the genotypic values are shown.

The gcc represents an integrated signal of canopy greenness. Until 70 DAS, the LAI increased to 6. Toward higher values, the scatterplots (Figure 7) show that the gcc signal saturated at an LAI of about 4.5. At low LAI (<1), the scatterplots indicate that there was no clear relationship between the LAI meter and gcc estimated LAI values. The LAI values of DAS 61 ranged from 0.35 to 2.93. In this span, the gcc corresponded well with the LAI on the phenotypic (*R*^2^ of 0.8) and genotypic level (*R*^2^ of 0.84). At DAS 40, a good relationship was found for plant vigor on the phenotypic level (*R*^2^ of 0.64) and the genotypic level (*R*^2^ of 0.78).

**FIGURE 8:**
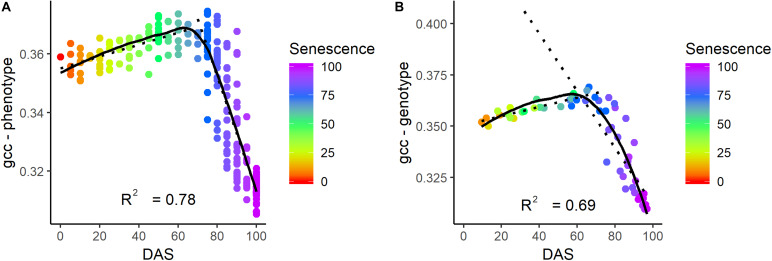
Relationship of senescence ratio to gcc on the phenotypic **(A)** and genotypic level **(B)** in days after sowing (DAS). The color corresponds to the senescence rating. The coefficient of determination (*R*^2^) is given for the relationship of gcc and senescence for a rating above 65%.

Visual senescence rating and gcc were not linearly related over time (Figure 8). While the visual rating constantly decreased over time, gcc showed a clear bimodal trend with a steady increase followed by a sharp decrease. Until a senescence rating of about 65%, there was a positive linear trend of gcc with the rating. After 65%, there was a strong negative trend (*R*^2^ of 0.78 and 0.69 on the phenotypic and genotypic level, respectively) of gcc and the rating. This also showed that the SP approximately corresponds to a senescence rating of 65%.

### PhenoTimePoints and PhenoPhases

Figure 4 shows the relation of the phenological signal to the manual rating of senescence and LAI of one plot: The LAI rapidly increased after SOG toward the GP. Around the GP, the LAI increase slowed. The senescence steeply increased toward the SP and reached 100% at EOS. The SP approximately corresponded to a senescence rating of 65%.

Figure 6 shows boxplots of the timings of the PhenoPhases and PhenoTimePoints. The timings of the PhenoTimePoint SOG only showed a small variability of 3 days. The GP, SP, and EOS showed greater variability (more than 7 days). The PhenoPhase SP_SOG showed a high range of durations (more than 14 days) being in the same order of magnitude as the maturation difference known for the selected genotypes (between 1 and 3 weeks). SP_SOG showed a high range of 14 days, while the difference in duration of the other PhenoPhases was between 6 and 7 days.

### Interaction Between Phenological Timing and Harvest Traits

With the information on the timing and duration of the PhenoTimePoints and PhenoPhases, it is possible to correlate these with harvest traits. In the following, we show the potential of the approach by discussing (a) how the early vigor corresponded to the timing of the PhenoTimePoints of the different genotypes and (b) how the timing of PhenoPhases corresponded to the harvest traits dry thousand kernel weight, oil content and pod yield.

**FIGURE 9:**
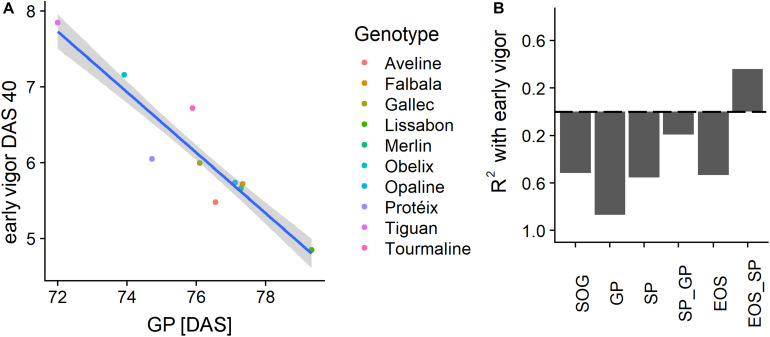
**(A)** Scatterplot of the early vigor rating at days after sowing (DAS) 40 with the timing of the green peak (GP) in DAS for the different genotypes. **(B)** Coefficient of determination (*R*^2^) of early vigor, PhenoTimePoints, and PhenoPhases. Bars below the dashed line correspond to negative correlations and vice versa. Results for the start of season are not shown due to the low range of the values.

Figure 9A shows a scatterplot of the vigor rating at DAS 40 to the timing of the GP of the different genotypes. A high rating of vigor strongly corresponded (*R*^2^ of 0.87) to an earlier GP. For the other PhenoTimePoints the relationship was moderate (Figure 9B). All relationships were negative, which indicated that a high early vigor generally led to earlier development. The PhenoPhases had a moderate to low (*R*^2^ < 0.4) relationship to the early vigor rating and were negative. Only the duration between SP and EOS had a moderate (*R*^2^ of 0.36) positive correlation to early vigor, which indicated that a better early growth prolongs senescence. Early vigor had no significant (*p* of 0.01) correlation to the total duration of growth after the GP peak was reached.

**FIGURE 10:**
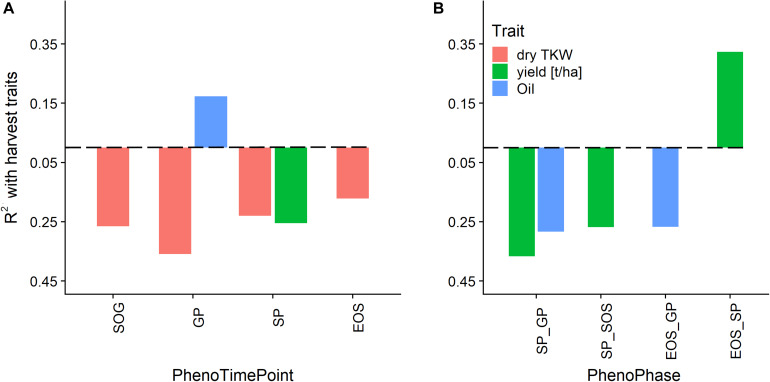
Relationship of phenological timing of PhenoTimePoints **(A)** and PhenoPhases **(B)** with harvest traits by means of coefficient of determination (*R*^2^). Bars below the dashed line correspond to negative correlations and vice versa. For readability, only PhenoPhases with an *R*^2^ of more than 0.25 are shown (all have a *p*-value of *<*0.01).

Figure 10 shows the correlation of the harvest traits yield, TKW, and oil content with the timings of the PhenoTimePoints and PhenoPhases. It revealed that varieties that had an earlier start of the green up phase and reached the GP earlier had a higher TKW. Varieties that reached the GP later had a higher oil content. Varieties that had a shorter green period (SP_GP) and an earlier SP had a higher yield, while varieties with a longer senescence period (EOS_SP) had a higher yield. Similarly, the oil content was higher when the duration between the GP and SP or EOS was longer. It has to be noted that these results might be specific for this experiment reflecting the genotype selection.

## Discussion

### Generation of the Phenological Signal

PhenoCam data are generally produced by uncalibrated cameras and are influenced by many factors. In this study, we identified (i) changing measurement geometry during the day, other (ii) changes in illumination conditions due to varying cloud cover, and (iii) the viewing geometry within an image as the main influences on the phenological signal. The gcc is known to suppress the effects of changes in scene illumination (Sonnentag et al., 2012). During initial tests, we could confirm that the gcc was more stable than other color indices (data not shown). To account for short term fluctuations of the signal (i, ii) we calculated the daily median to normalize for the diurnal differences and used the Savitzky-Golay filter to buffer day-to-day variations resulting from short term differences due to changing weather conditions. The approach showed to be more successful than other approaches (e.g., 90th percentile) to derive a smooth signal across the season. Still, two uncertainties remain: With low crop cover, the soil background affects the signal. Wet periods of several days reduce the gcc value as seen around DAS 25. Also, plant canopies appear differently in direct and diffuse illumination due to the varied shading within the canopy (Aasen and Bolten, 2018), which is still an open problem in remote sensing and field phenotyping (e.g., Yu et al., 2017) and hard to address in a general manner without actual information on canopy structure. Nevertheless, the daily mean in combination with the Savitzky-Golay filter seemed to normalize fluctuating illumination conditions quite well. The different pixel count per plot, which results from different distances to the camera, can be assumed to have negligible effect on the signal, since pixels covering a larger area represent the average signal of this area. Still, a sufficient resolution is required for cameras to ensure that pixels potentially contaminated from border effects can be excluded.

The systematic bias resulting from viewing geometry (iii) could be mitigated by the spatial correction approach along with potential in-field heterogeneity. The gcc at most DAS and most timings of the PhenoTimePoints were influenced by the oblique viewing geometry (Figure 5), as has also been reported for LAI in forest environments (Keenan et al., 2014). The main reason for this is that the zenith angle interacts with canopy structure, which results in occlusion effects (Aasen and Bolten, 2018; Roth et al., 2018). Considering the pattern caused by the viewing geometry (Figure 2B) as observational bias during the spatial correction could remove the trend (Figure 5) and most of the remaining variance could be attributed to the differences between the genotypes as demonstrated by the high heritability (Figure 6). The drawback of this approach is that it relies on an experimental design that is suited for spatial correction procedures, such as those implemented in SpATS or similar approaches. Still, the viewing geometries mainly influenced the absolute values of the gcc. In comparison, the timing of the PhenoTimePoints was barely influenced (Figure 5 and Supplementary Table S3). Thus, two cases can be distinguished: (a) when the gcc should be used as a proxy for a trait (e.g., LAI and vigor), a spatial correction seems to be necessary; and (b) when the gcc dynamics are used to estimate PhenoTimePoints it does not seem to be absolutely necessary to apply a spatial correction. Regarding the absolute values, it also has to be noted that these results will mainly depend on the differences between the viewing geometries of each plot. When a camera is mounted further away from the experiment, the viewing geometries for each plot will be more similar and, consequently, will have less impact on the results. Additionally, the absolute influence of the viewing geometry will always depend on the canopy structure and thus differ between crops. Besides, further studies could advance the spatial correction approach by separating the effects resulting from the viewing geometry from field-heterogeneity effects.

### Extraction of Traits, PhenoTimePoints, and PhenoPhases

The analysis showed that the gcc could be used to estimate LAI between approximately 1 and 4.5. Higher LAI values were not detectable due to saturation of the gcc resulting from the mentioned occlusion effects in combination with almost full canopy cover. The inaccuracies of lower LAI values likely result from the inaccuracies from our reference method. It is known that the Plant Canopy Analyzer (LAI-2200) cannot capture low LAI (<1) values reliably in row crops (c.f. Roth et al., 2018). Since crop growth is most rapid for LAI 1 to 4.5 (e.g., Figure 4), capturing the development in this phase is already a benefit for many applications in breeding and variety testing. It is likely that the exact relationships between gcc and LAI are crop and site specific (e.g., in brighter soils also lower LAI values will be visible) and will be influenced by the sowing density and row orientation. Nevertheless, the general concept is broadly applicable. Future studies should investigate the relationships for other crops and sowing patterns.

We could identify four points within the phenological signal that could be attributed to phenological events, namely the start of green up (SOG), green peak (GP), senescence peak (SP), and end of senescence/end of season (EOS). Different terminologies have been used in the ecological literature, and we tried to align with the existing terminology. In the case of SOG, we refrain from calling it start of season, since the plants did already grow before, but our model could not reliably resolve it (see below). The GP and SP are uncommon in the ecological literature. But, the analysis showed that these PhenoTimePoints can be attributed to quantitative ratings for LAI and senescence of soybean. Figure 7 shows that the maximum value of the gcc is around an LAI of 4.5, and consequently, the peak during green up (GP) can be attributed to this value. Figure 8 shows that the gcc reaches a maximum during senescence at a rating of 65%, and consequently, the peak of gcc during the senescence phase (SP) can be attributed to this value. As previously mentioned, it has to be noted that it is likely that these values are species specific because they are influenced by canopy structure. Also, it has to be mentioned that senescence in field crops often starts at leaf levels close to the ground. This is a shortcoming of the presented method since oblique images only capture the top of canopy. This also needs to be taken into account when setting up a camera, since in crops where the canopy is not 100% closed at the time of senescence—such as cereals—viewing geometries close to nadir will allow seeing into the canopy and thus, possibly capture processes earlier than in parts of an image with oblique viewing geometries. Thus, ideally, a camera should be placed in some distance to the experiment such that the viewing geometry differences are small. Finally, the method to extract the PhenoTimePoints relies on significant changes in the slope of the gcc. This turned out to be a shortcoming in extracting the point for start of senescence, which did not work reliably for all genotypes. We ascribe that to the fact that some plots already started a very slow yellowing process around DAS 99, which did not produce a pronounced slope change (Supplementary Figure S1).

### Investigating Interactions of Growth, Harvest Traits and Phenology With PhenoCams

While there is a conceptual understanding on how phenology may be influenced by climate conditions, it is challenging to provide quantitative estimates of the magnitude of these shifts (Richardson et al., 2013; Herrera et al., 2018). Field phenotyping with PhenoCams is a low cost, fully automated approach to gather very high temporal resolution data of an area sufficient for experimental trials. We could show that such an approach is particularly useful to monitor (i) rapid growth dynamics that might only differ by or last a few days, (ii) investigate differences in phenology across genotypes, and (iii) analyze the interactions between crop development (e.g., early vigor, LAI, and green up dynamics), phenology (e.g., senescence dynamics) and harvest traits.

With the highly temporal-resolved data, we could investigate the influence of early vigor on subsequent PhenoTimePoints and duration of PhenoPhases. While the focus of this manuscript is on the methodology, the obtained results are plausible. Early vigor is positively correlated with GP showing the faster canopy closure of more vigorous genotypes. Similar observations were reported using canopy cover measures instead of GP (Kipp et al., 2014; Kirchgessner et al., 2017). Besides, we found that yield and yield-quality traits such as oil content and TKW are related to a longer green period, late and short senescence, which has previously been reported to correspond to higher yield performance under normal or stress situations (Kropff et al., 1993; Thomas and Smart, 1993; De Souza et al., 1997; Rebetzke et al., 2016). While these interactions are not new knowledge *per se*, so far it was very hard to quantify these relationships for multiple genotypes in the field. Because these insights are connected to differences between genotypes, analyzing datasets of multiple years would also allow assessing, e.g., the impact of climate-induced phenological shifts.

### Comparison to Other Approaches

PhenoCam data allows monitoring multiple areas of interest at the same time (e.g., Julitta et al., 2014; Browning et al., 2017; this study) in very high temporal resolution. As shown, they can monitor various types of traits across the whole season with minimal effort. Still, the approach is limited to some basic traits that are observable with the spatial ground sampling distance of the cameras. Besides, the approach is not suitable to estimate biochemical traits due to the missing radiometric calibration and spectral characterization. While encouraging approaches exist, e.g., using very high-resolution gray card calibrated nadir images along with plant segmentation to estimate leaf nitrogen concentration levels (Wang et al., 2014), the bandwidth of usual RGB cameras are typically too wide to fit the absorption features of e.g., pigments. Continuous spectral measurement systems may be superior for that purpose (e.g., D’Odorico et al., 2015; Paul-Limoges et al., 2018; Wieneke et al., 2018). However, temporally continuous spectral measurements are mostly limited to point measurements (Aasen et al., 2019).

For a field phenotyping scenario compared to the one of this study, where a couple of hundred plots (depending on the plot size) on an area of approximately 2 ha are to be screened for basic phenological parameters, the PhenoCam approach has lower operational and setup cost, higher temporal resolution but lower precision compared to (more or less) mobile platforms that monitor the canopy from close range (e.g., Cendrero-Mateo et al., 2017; Kirchgessner et al., 2017; Hund et al., 2019). Flying mobile platforms (UAV) that monitor the canopy from a distance of a couple of decameters with RGB sensors are quite flexible, can be used to monitor phenology (Burkart et al., 2018) and can even be used to track the green up based on plant segmentation (e.g., Roth et al., 2018). But while RGB flights are almost always possible with a high temporal resolution (Aasen and Bareth, 2018; Roth et al., 2018), each flight campaign is connected with some effort, because fully autonomous systems are currently prohibited by legislation in most countries. Generally, mobile platforms can cover a larger area. Close-range PhenoCams that are mounted right above the canopy could combine the benefits of both approaches but would be very costly to set up. Table 3 summarizes these considerations. Looking forward, one could imagine a network of multiple PhenoCams that cover larger trials, even across different environments and for multiple years. In the end, many traits need to be captured by field phenotyping approaches to complement each other in order to get the full picture. PhenoCams can become an important tool providing high temporal resolution information on phenological processes.

**TABLE 3:** Comparison of different approaches to measure basic phenological parameters.

	Close range mobile systems	UAVs	Field PhenoCam	Close range PhenoCams
Cost	−−	0	++	−
Set up effort	−−	−	+	−
Operational effort	−−	−	++	++
Spatial resolution	++	+	−−	++
Realistic temporal resolution	0	+	++	++

*+ and ++, advantages; − and −−, disadvantages.*

## Data Availability Statement

The datasets generated for this study are available on request to the corresponding author.

## Author Contributions

HA developed the idea, conceived the study together with FL, analyzed the data, and wrote large parts of the manuscript. NK operated the PhenoCam as part of the FIP system, provided the technical details, and supported the data analysis. FL organized and conducted the field experiment and supported the data analysis. HA, NK, AW, and FL contributed to writing of the manuscript.

## Conflict of Interest

The authors declare that the research was conducted in the absence of any commercial or financial relationships that could be construed as a potential conflict of interest.

## Footnotes

1 https://www.lupus-electronics.de

## References

[B1] Aasen H. Bareth G. (2018). “Ground and UAV sensing approaches for spectral and 3D crop trait estimation,” in *Hyperspectral Remote Sensing of Vegetation - Volume II: Biophysical and Biochemical Characterization and Plant Species Studies*, eds Thenkabail P. Lyon J. G. Huete A. (Milton: Chapman and Hall).

[B2] Aasen H. Bolten A. (2018). Multi-temporal high-resolution imaging spectroscopy with hyperspectral 2D imagers – From theory to application. *Remote Sens. Environ.* 205 374–389. 10.1016/j.rse.2017.10.043

[B3] Aasen H. Honkavaara E. Lucieer A. Zarco-Tejada P. (2018). Quantitative remote sensing at ultra-high resolution with UAV spectroscopy: a review of sensor technology, measurement procedures, and data correction workflows. *Remote Sens.* 10:1091 10.3390/rs10071091

[B4] Aasen H. Van Wittenberghe S. Medina N. S. Damm A. Goulas Y. Wieneke S. (2019). Sun-induced Chlorophyll Fluorescence ii: review of passive measurement setups, protocols, and their application at the leaf to canopy level. *Remote Sens.* 11:927 10.3929/ethz-b-000340341

[B5] Ahrends H. Etzold S. Kutsch W. Stoeckli R. Bruegger R. Jeanneret F. (2009). Tree phenology and carbon dioxide fluxes: use of digital photography for process-based interpretation at the ecosystem scale. *Clim. Res.* 39 261–274. 10.3354/cr00811

[B6] Anderegg J. Yu K. Aasen H. Walter A. Liebisch F. Hund A. (2020). Spectral vegetation indices to track senescence dynamics in diverse wheat Germplasm. *Front. Plant Sci.* 10:1749. 10.3389/fpls.2019.01749 32047504 PMC6997566

[B7] Anderson H. Nilsen L. Tømmervik H. Karlsen S. Nagai S. Cooper E. (2016). Using ordinary digital cameras in place of near-infrared sensors to derive vegetation indices for phenology studies of high arctic vegetation. *Remote Sens.* 8:847 10.3390/rs8100847

[B8] Andresen C. G. Tweedie C. E. Lougheed V. L. (2018). Climate and nutrient effects on Arctic wetland plant phenology observed from phenocams. *Remote Sens. Environ.* 205 46–55. 10.1016/j.rse.2017.11.013

[B9] Araus J. L. Kefauver S. C. Zaman-Allah M. Olsen M. S. Cairns J. E. (2018). Translating high-throughput phenotyping into genetic gain. *Trends Plant Sci.* 23 451–466. 10.1016/j.tplants.2018.02.001 29555431 PMC5931794

[B10] Asseng S. Ewert F. Martre P. Rötter R. P. Lobell D. B. Cammarano D. (2015). Rising temperatures reduce global wheat production. *Nat. Clim. Change* 5 143–147. 10.1038/nclimate2470

[B11] Asseng S. Ewert F. Rosenzweig C. Jones J. W. Hatfield J. L. Ruane A. C. (2013). Uncertainty in simulating wheat yields under climate change. *Nat. Clim. Change* 3 827–832. 10.1038/nclimate1916

[B12] Bendig J. Yu K. Aasen H. Bolten A. Bennertz S. Broscheit J. (2015). Combining UAV-based plant height from crop surface models, visible, and near infrared vegetation indices for biomass monitoring in barley. *Int. J. Appl. Earth Obs. Geoinform.* 39 79–87. 10.1016/j.jag.2015.02.012

[B13] Brown T. B. Hultine K. R. Steltzer H. Denny E. G. Denslow M. W. Granados J. (2016). Using phenocams to monitor our changing Earth: toward a global phenocam network. *Front. Ecol. Environ.* 14:84–93. 10.1002/fee.1222

[B14] Browning D. Karl J. Morin D. Richardson A. Tweedie C. (2017). Phenocams bridge the gap between field and satellite observations in an arid grassland ecosystem. *Remote Sens.* 9:1071 10.3390/rs9101071

[B15] Burkart A. Hecht V. L. Kraska T. Rascher U. (2018). Phenological analysis of unmanned aerial vehicle based time series of barley imagery with high temporal resolution. *Precis. Agric.* 19 134–146. 10.1007/s11119-017-9504-y

[B16] Busemeyer L. Mentrup D. Möller K. Wunder E. Alheit K. Hahn V. (2013). BreedVision — a multi-sensor platform for non-destructive field-based phenotyping in plant breeding. *Sensors* 13 2830–2847. 10.3390/s130302830 23447014 PMC3658717

[B17] Cao R. Chen Y. Shen M. Chen J. Zhou J. Wang C. (2018). A simple method to improve the quality of NDVI time-series data by integrating spatiotemporal information with the Savitzky-Golay filter. *Remote Sens. Environ.* 217 244–257. 10.1016/j.rse.2018.08.022

[B18] Cendrero-Mateo M. P. Muller O. Albrecht H. Burkart A. Gatzke S. Janssen B. (2017). “Field phenotyping: concepts and examples to quantify dynamic plant traits across scales in the field,” in *Terrestrial Ecosystem Research Infrastructures: Challenges and Opportunities Abad Chabbi*, eds Chabbi A. Loescher H. W. (Boca Raton: Abad Chabbi, Henry W. Loescher; CRC Press), 53–80.

[B19] Chen J. Jönsson P. Tamura M. Gu Z. Matsushita B. Eklundh L. (2004). A simple method for reconstructing a high-quality NDVI time-series data set based on the Savitzky–Golay filter. *Remote Sens. Environ.* 91 332–344. 10.1016/j.rse.2004.03.014

[B20] De Souza P. I. Egli D. B. Bruening W. P. (1997). Water stress during seed filling and leaf senescence in Soybean. *Agron. J.* 89 807 10.2134/agronj1997.00021962008900050015x

[B21] Deery D. Jimenez-Berni J. Jones H. Sirault X. Furbank R. (2014). Proximal remote sensing buggies and potential applications for field-based phenotyping. *Agronomy* 4 349–379. 10.3390/agronomy4030349

[B22] D’Odorico P. Gonsamo A. Gough C. M. Bohrer G. Morison J. Wilkinson M. (2015). The match and mismatch between photosynthesis and land surface phenology of deciduous forests. *Agric. For. Meteorol.* 21 25–38. 10.1016/j.agrformet.2015.07.005

[B23] Donnelly A. Yu R. Caffarra A. Hanes J. Liang L. Desai A. R. (2017). Interspecific and interannual variation in the duration of spring phenophases in a northern mixed forest. *Agric. For. Meteorol.* 243 55–67. 10.1016/j.agrformet.2017.05.007

[B24] Fan X. Kawamura K. Guo W. Xuan T. D. Lim J. Yuba N. (2018). A simple visible and near-infrared (V-NIR) camera system for monitoring the leaf area index and growth stage of Italian ryegrass. *Comput. Electron. Agric.* 144 314–323. 10.1016/j.compag.2017.11.025

[B25] Filippa G. Cremonese E. Migliavacca M. Galvagno M. Forkel M. Wingate L. (2016). Phenopix: a R package for image-based vegetation phenology. *Agric. Forest Meteorol.* 220 141–150. 10.1016/j.agrformet.2016.01.006

[B26] Filippa G. Cremonese E. Migliavacca M. Galvagno M. Sonnentag O. Humphreys E. (2018). NDVI derived from near-infrared-enabled digital cameras: applicability across different plant functional types. *Agric. Forest Meteorol.* 249 275–285. 10.1016/j.agrformet.2017.11.003

[B27] Fiorani F. Schurr U. (2013). Future scenarios for plant phenotyping. *Annu. Rev. Plant Biol.* 64 267–291. 10.1146/annurev-arplant-050312-120137 23451789

[B28] Gillespie A. R. Kahle A. B. Walker R. E. (1987). Color enhancement of highly correlated images. II. Channel ratio and “chromaticity” transformation techniques. *Remote Sens. Environ.* 22 343–365. 10.1016/0034-4257(87)90088-5

[B29] Gilmour A. R. Cullis B. R. Verbyla A. P. Verbyla A. P. (1997). Accounting for natural and extraneous variation in the analysis of field experiments. *J. Agric. Biol. Environ. Stat.* 2:269 10.2307/1400446

[B30] Gnyp M. L. Yu K. Aasen H. Yao Y. Huang S. Miao Y. (2013). Analysis of crop reflectance for estimating biomass in rice canopies at different phenological stages. *Photogramm. Fernerkund. Geoinform.* 2013 351–365. 10.1127/1432-8364/2013/0182

[B31] Gómez-Candón D. Virlet N. Labbé S. Jolivot A. Regnard J.-L. (2016). Field phenotyping of water stress at tree scale by UAV-sensed imagery: new insights for thermal acquisition and calibration. *Precis. Agric.* 17 786–800. 10.1007/s11119-016-9449-6

[B32] Graham E. A. Riordan E. C. Yuen E. M. Estrin D. Rundel P. W. (2010). Public Internet-connected cameras used as a cross-continental ground-based plant phenology monitoring system: public cameras as phenology monitoring system. *Glob. Change Biol.* 16 3014–3023. 10.1111/j.1365-2486.2010.02164.x

[B33] Haghighattalab A. González Pérez L. Mondal S. Singh D. Schinstock D. Rutkoski J. (2016). Application of unmanned aerial systems for high throughput phenotyping of large wheat breeding nurseries. *Plant Methods* 12:35. 10.1186/s13007-016-0134-6 27347001 PMC4921008

[B34] Herrera J. M. Häner L. L. Holzkämper A. Pellet D. (2018). Evaluation of ridge regression for country-wide prediction of genotype-specific grain yields of wheat. *Agric. Forest Meteorol.* 252 1–9. 10.1016/j.agrformet.2017.12.263

[B35] Hu P. Chapman S. C. Wang X. Potgieter A. Duan T. Jordan D. (2018). Estimation of plant height using a high throughput phenotyping platform based on unmanned aerial vehicle and self-calibration: example for sorghum breeding. *Eur. J. Agron.* 95 24–32. 10.1016/j.eja.2018.02.004

[B36] Hufkens K. Basler D. Milliman T. Melaas E. K. Richardson A. D. (2018a). An integrated phenology modelling framework in R. *Methods Ecol. Evol.* 9 1276–1285. 10.1111/2041-210X.12970

[B37] Hufkens K. Filippa G. Cremonese E. Migliavacca M. D’Odorico P. Peichl M. (2018b). Assimilating phenology datasets automatically across ICOS ecosystem stations. *Int. Agrophys.* 32 677–687. 10.1515/intag-2017-0050

[B38] Hufkens K. Friedl M. Sonnentag O. Braswell B. H. Milliman T. Richardson A. D. (2012). Linking near-surface and satellite remote sensing measurements of deciduous broadleaf forest phenology. *Remote Sens. Environ.* 117 307–321. 10.1016/j.rse.2011.10.006

[B39] Hufkens K. Keenan T. F. Flanagan L. B. Scott R. L. Bernacchi C. J. Joo E. (2016). Productivity of North American grasslands is increased under future climate scenarios despite rising aridity. *Nat. Clim. Change* 6 710–714. 10.1038/nclimate2942

[B40] Hund A. Kronenberg L. Anderegg J. Yu K. Walter A. (2019). “Non-invasive phenotyping of cereal growth and development characteristics in the field,” in *Advances in Crop Breeding Techniques*, eds Ordon F. Friedt W. (Burleigh Dodds), 10.19103/AS.2019.0051.13

[B41] Ide R. Oguma H. (2010). Use of digital cameras for phenological observations. *Ecol. Inform.* 5 339–347. 10.1016/j.ecoinf.2010.07.002

[B42] Jimenez-Berni J. A. Deery D. M. Rozas-Larraondo P. Condon A. Tony G. Rebetzke G. J. (2018). High throughput determination of plant height, ground cover, and above-ground biomass in wheat with LiDAR. *Front. Plant Sci.* 9:237. 10.3389/fpls.2018.00237 29535749 PMC5835033

[B43] Jönsson P. Eklundh L. (2004). TIMESAT—a program for analyzing time-series of satellite sensor data. *Comput. Geosci.* 30 833–845. 10.1016/j.cageo.2004.05.006

[B44] Julitta T. Cremonese E. Migliavacca M. Colombo R. Galvagno M. Siniscalco C. (2014). Using digital camera images to analyse snowmelt and phenology of a subalpine grassland. *Agric. Forest Meteorol.* 19 116–125. 10.1016/j.agrformet.2014.08.007

[B45] Keenan T. F. Darby B. Felts E. Sonnentag O. Friedl M. A. Hufkens K. (2014). Tracking forest phenology and seasonal physiology using digital repeat photography: a critical assessment. *Ecol. Appl.* 24 1478–1489. 10.1890/13-0652.1 29160668

[B46] Keenan T. F. Richardson A. D. (2015). The timing of autumn senescence is affected by the timing of spring phenology: implications for predictive models. *Glob. Change Biol.* 21 2634–2641. 10.1111/gcb.12890 25662890

[B47] Kefauver S. C. Vicente R. Vergara-Díaz O. Fernandez-Gallego J. A. Kerfal S. Lopez A. (2017). Comparative UAV and field phenotyping to assess yield and Nitrogen use efficiency in hybrid and conventional barley. *Front. Plant Sci.* 8:1733. 10.3389/fpls.2017.01733 29067032 PMC5641326

[B48] Kipp S. Mistele B. Baresel P. Schmidhalter U. (2014). High-throughput phenotyping early plant vigour of winter wheat. *Eur. J. Agron.* 52 271–278. 10.1016/j.eja.2013.08.009

[B49] Kirchgessner N. Liebisch F. Yu K. Pfeifer J. Friedli M. Hund A. (2017). The ETH field phenotyping platform FIP: a cable-suspended multi-sensor system. *Funct. Plant Biol.* 44 154. 10.1071/FP16165 32480554

[B50] Klosterman S. T. Hufkens K. Gray J. M. Melaas E. Sonnentag O. Lavine I. (2014). Evaluating remote sensing of deciduous forest phenology at multiple spatial scales using PhenoCam imagery. *Biogeosciences* 11 4305–4320. 10.5194/bg-11-4305-2014

[B51] Kropff M. J. Cassman K. G. Peng S. Matthew R. B. Setter T. L. (1993). “Quantitative understanding of yield potential,” in *Breaking the Yield Barrier: Proceedings of a Workshop on Rice Yield Potential in Favorable Environments IRRI*, ed. Cassman K. G. (Los Banos: IRRI), 141.

[B52] Kurc S. A. Benton L. M. (2010). Digital image-derived greenness links deep soil moisture to carbon uptake in a creosotebush-dominated shrubland. *J. Arid Environ.* 74 585–594. 10.1016/j.jaridenv.2009.10.003

[B53] Li L. Zhang Q. Huang D. (2014). A review of imaging techniques for plant phenotyping. *Sensors* 14 20078–20111. 10.3390/s141120078 25347588 PMC4279472

[B54] Liebisch F. Kirchgessner N. Schneider D. Walter A. Hund A. (2015). Remote, aerial phenotyping of maize traits with a mobile multi-sensor approach. *Plant Methods* 11:9. 10.1186/s13007-015-0048-8 25793008 PMC4365514

[B55] Liu Y. Hill M. J. Zhang X. Wang Z. Richardson A. D. Hufkens K. (2017). Using data from Landsat, MODIS, VIIRS and PhenoCams to monitor the phenology of California oak/grass savanna and open grassland across spatial scales. *Agric. For. Meteorol.* 23 311–325. 10.1016/j.agrformet.2017.02.026

[B56] Liu Z. An S. Lu X. Hu H. Tang J. (2018). Using canopy greenness index to identify leaf ecophysiological traits during the foliar senescence in an oak forest. *Ecosphere* 9:e02337 10.1002/ecs2.2337

[B57] Madec S. Baret F. de Solan B. Thomas S. Dutartre D. Jezequel S. (2017). High-throughput phenotyping of plant height: comparing unmanned aerial vehicles and ground LiDAR Estimates. *Front. Plant Sci.* 8:2002. 10.3389/fpls.2017.02002 29230229 PMC5711830

[B58] Migliavacca M. Galvagno M. Cremonese E. Rossini M. Meroni M. Sonnentag O. (2011). Using digital repeat photography and eddy covariance data to model grassland phenology and photosynthetic CO2 uptake. *Agric. For. Meteorol.* 151 1325–1337. 10.1016/j.agrformet.2011.05.012

[B59] Molenaar H. Boehm R. Piepho H.-P. (2018). Phenotypic selection in ornamental breeding: it’s better to have the BLUPs than to have the BLUEs. *Front. Plant Sci.* 9:1511. 10.3389/fpls.2018.01511 30455707 PMC6230591

[B60] Munger P. Bleiholder H. Hack H. Hess M. Stauss R. Boom T. (1997). Phenological growth stages of the soybean plant (Glycine max L. MERR.): codification and description according to the BBCH Scale. *J. Agron. Crop Sci.* 179 209–217. 10.1111/j.1439-037X.1997.tb00519.x

[B61] Nagai S. Akitsu T. Saitoh T. M. Busey R. C. Fukuzawa K. Honda Y. (2018). 8 million phenological and sky images from 29 ecosystems from the Arctic to the tropics: the Phenological Eyes Network. *Ecol. Res.* 33 1091–1092. 10.1007/s11284-018-1633-x

[B62] Paul-Limoges E. Damm A. Hueni A. Liebisch F. Eugster W. Schaepman M. E. (2018). Effect of environmental conditions on sun-induced fluorescence in a mixed forest and a cropland. *Remote Sens. Environ.* 219 310–323. 10.1016/j.rse.2018.10.018

[B63] Penuelas J. Rutishauser T. Filella I. (2009). Phenology feedbacks on climate change. *Science* 324 887–888. 10.1126/science.1173004 19443770

[B64] Peter R. Eschholz T. W. Stamp P. Liedgens M. (2009). Swiss Flint maize landraces—A rich pool of variability for early vigour in cool environments. *Field Crops Res.* 110 157–166. 10.1016/j.fcr.2008.07.015

[B65] Piepho H. P. Möhring J. Melchinger A. E. Büchse A. (2008). BLUP for phenotypic selection in plant breeding and variety testing. *Euphytica* 161 209–228. 10.1007/s10681-007-9449-8

[B66] Piepho H. P. Williams E. R. (2010). Linear variance models for plant breeding trials. *Plant Breed.* 129 1–8. 10.1002/bimj.200710414 18383446

[B67] Pieruschka R. (2016). *Plant Phenotyping Survey 2016.* Available online at: https://www.plant-phenotyping.org/ippn-survey_2016 (accessed October 18, 2018).

[B68] Pretty J. Sutherland W. J. Ashby J. Auburn J. Baulcombe D. Bell M. (2010). The top 100 questions of importance to the future of global agriculture. *Int. J. Agric. Sustain.* 8 219–236. 10.3763/ijas.2010.0534

[B69] Rebetzke G. J. Jimenez-Berni J. A. Bovill W. D. Deery D. M. James R. A. (2016). High-throughput phenotyping technologies allow accurate selection of stay-green. *J. Exp. Bot.* 67 4919–4924. 10.1093/jxb/erw301 27604804 PMC5014170

[B70] Reid A. M. Chapman W. K. Prescott C. E. Nijland W. (2016). Using excess greenness and green chromatic coordinate colour indices from aerial images to assess lodgepole pine vigour, mortality and disease occurrence. *Forest Ecol. Manag.* 374 146–153. 10.1016/j.foreco.2016.05.006

[B71] Richardson A. D. (2019). Tracking seasonal rhythms of plants in diverse ecosystems with digital camera imagery. *New Phytol.* 222 1742–1750. 10.1111/nph.15591 30415486

[B72] Richardson A. D. Braswell B. H. Hollinger D. Y. Jenkins J. P. Ollinger S. V. (2009). Near-surface remote sensing of spatial and temporal variation in canopy phenology. *Ecol. Appl.* 19 1417–1428. 10.1890/08-2022.1 19769091

[B73] Richardson A. D. Hufkens K. Milliman T. Aubrecht D. M. Chen M. Gray J. M. (2018a). Tracking vegetation phenology across diverse North American biomes using PhenoCam imagery. *Sci. Data* 5:180028. 10.1038/sdata.2018.28 29533393 PMC5848786

[B74] Richardson A. D. Hufkens K. Milliman T. Aubrecht D. M. Furze M. E. Seyednasrollah B. (2018b). Ecosystem warming extends vegetation activity but heightens vulnerability to cold temperatures. *Nature* 560 368–371. 10.1038/s41586-018-0399-1 30089905

[B75] Richardson A. D. Jenkins J. P. Braswell B. H. Hollinger D. Y. Ollinger S. V. Smith M.-L. (2007). Use of digital webcam images to track spring green-up in a deciduous broadleaf forest. *Oecologia* 152 323–334. 10.1007/s00442-006-0657-z 17342508

[B76] Richardson A. D. Keenan T. F. Migliavacca M. Ryu Y. Sonnentag O. Toomey M. (2013). Climate change, phenology, and phenological control of vegetation feedbacks to the climate system. *Agric. Forest Meteorol.* 169 156–173. 10.1016/j.agrformet.2012.09.012

[B77] Rodríguez-Álvarez M. X. Boer M. P. van Eeuwijk F. A. Eilers P. H. C. (2018). Correcting for spatial heterogeneity in plant breeding experiments with P-splines. *Spat. Stat.* 23 52–71. 10.1016/j.spasta.2017.10.003

[B78] Roth L. Aasen H. Walter A. Liebisch F. (2018). Extracting leaf area index using viewing geometry effects—A new perspective on high-resolution unmanned aerial system photography. *ISPRS J. Photogramm. Remote Sens.* 141 161–175. 10.1016/j.isprsjprs.2018.04.012

[B79] Sakamoto T. Gitelson A. A. Nguy-Robertson A. L. Arkebauer T. J. Wardlow B. D. Suyker A. E. (2012). An alternative method using digital cameras for continuous monitoring of crop status. *Agric. For. Meteorol.* 154–155 113–126. 10.1016/j.agrformet.2011.10.014

[B80] Savitzky A. Golay M. J. E. (1964). Smoothing and differentiation of data by simplified least squares procedures. *Anal. Chem.* 36 1627–1639. 10.1021/ac60319a045 22324618

[B81] Schwärzel R. Hiltbrunner J. (2015). Liste der empfohlenen Sojasorten für die Ernte. *Agrarforsch. Schweiz* 6 Available online at: https://www.agrarforschungschweiz.ch/2015/02/liste-der-empfohlenen-sojasorten-fuer-die-ernte-2015/

[B82] Brocks S. Bareth G. (2018). Estimating barley biomass with crop surface models from oblique RGB imagery. *Remote Sens.* 10:268 10.3390/rs10020268

[B83] Siebert S. Ewert F. (2014). Future crop production threatened by extreme heat. *Environ. Res. Lett.* 9:041001 10.1088/1748-9326/9/4/041001

[B84] Snyder K. Wehan B. Filippa G. Huntington J. Stringham T. Snyder D. (2016). Extracting plant phenology metrics in a great basin watershed: methods and considerations for quantifying phenophases in a cold desert. *Sensors* 16:1948. 10.3390/s16111948 27869752 PMC5134607

[B85] Sonnentag O. Hufkens K. Teshera-Sterne C. Young A. M. Friedl M. Braswell B. H. (2012). Digital repeat photography for phenological research in forest ecosystems. *Agric. For. Meteorol.* 152 159–177. 10.1016/j.agrformet.2011.09.009

[B86] Thomas H. Smart C. M. (1993). Crops that stay green. *Ann. Appl. Biol.* 123 193–219. 10.1111/j.1744-7348.1993.tb04086.x

[B87] Tilly N. Aasen H. Bareth G. (2015). Fusion of plant height and vegetation indices for the estimation of barley biomass. *Remote Sens.* 7 11449–11480. 10.3390/rs70911449

[B88] Toda M. Richardson A. D. (2018). Estimation of plant area index and phenological transition dates from digital repeat photography and radiometric approaches in a hardwood forest in the Northeastern United States. *Agric. For. Meteorol.* 249 457–466. 10.1016/j.agrformet.2017.09.004

[B89] Velazco J. G. Rodríguez-Álvarez M. X. Boer M. P. Jordan D. R. Eilers P. H. C. Malosetti M. (2017). Modelling spatial trends in sorghum breeding field trials using a two-dimensional P-spline mixed model. *Theor. Appl. Genet.* 130 1375–1392. 10.1007/s00122-017-2894-4 28374049 PMC5487705

[B90] Virlet N. Sabermanesh K. Sadeghi-Tehran P. Hawkesford M. J. (2017). Field Scanalyzer: an automated robotic field phenotyping platform for detailed crop monitoring. *Funct. Plant Biol.* 44 143. 10.1071/FP16163 32480553

[B91] Walter A. Liebisch F. Hund A. (2015). Plant phenotyping: from bean weighing to image analysis. *Plant Methods* 11:14. 10.1186/s13007-015-0056-8 25767559 PMC4357161

[B92] Wang Y. Wang D. Shi P. Omasa K. (2014). Estimating rice chlorophyll content and leaf nitrogen concentration with a digital still color camera under natural light. *Plant Methods* 10:36. 10.1186/1746-4811-10-36 25411579 PMC4236477

[B93] White J. W. Andrade-Sanchez P. Gore M. A. Bronson K. F. Coffelt T. A. Conley M. M. (2012). Field-based phenomics for plant genetics research. *Field Crops Res.* 133 101–112. 10.1016/j.fcr.2012.04.003

[B94] Wieneke S. Burkart A. Cendrero-Mateo M. P. Julitta T. Rossini M. Schickling A. (2018). Linking photosynthesis and sun-induced fluorescence at sub-daily to seasonal scales. *Remote Sens. Environ.* 219 247–258. 10.1016/j.rse.2018.10.019

[B95] Xie Y. Civco D. L. Silander J. A. (2018). Species-specific spring and autumn leaf phenology captured by time-lapse digital cameras. *Ecosphere* 9:e02089 10.1002/ecs2.2089

[B96] Yang G. Liu J. Zhao C. Li Z. Huang Y. Yu H. (2017). Unmanned aerial vehicle remote sensing for field-based crop phenotyping: current status and perspectives. *Front. Plant Sci.* 8:1111. 10.3389/fpls.2017.01111 28713402 PMC5492853

[B97] Yu K. Kirchgessner N. Grieder C. Walter A. Hund A. (2017). An image analysis pipeline for automated classification of imaging light conditions and for quantification of wheat canopy cover time series in field phenotyping. *Plant Methods* 13 1–13. 10.1186/s13007-017-0168-4 28344634 PMC5361853

[B98] Zaman-Allah M. Vergara O. Araus J. L. Tarekegne A. Magorokosho C. Zarco-Tejada P. J. (2015). Unmanned aerial platform-based multi-spectral imaging for field phenotyping of maize. *Plant Methods* 11:35. 10.1186/s13007-015-0078-2 26106438 PMC4477614

[B99] Zhu Y. Cao Z. Lu H. Li Y. Xiao Y. (2016). In-field automatic observation of wheat heading stage using computer vision. *Biosyst. Eng.* 143 28–41. 10.1016/j.biosystemseng.2015.12.015

