# Supplementary material for: PhenoCams for Field Phenotyping: Using Very High Temporal Resolution Digital Repeated Photography to Investigate Interactions of Growth, Phenology, and Harvest Traits
Source: Front Plant Sci. 2020 Jun 18;11:593. doi: 10.3389/fpls.2020.00593 (PMC7314959; doi:10.3389/fpls.2020.00593)
Supplement: Supplementary file 1 [file Data_Sheet_1.docx]

**Article title: PhenoCams for field phenotyping: Using very high temporal resolution digital repeated photography to investigate interactions of growth, phenology and harvest traits**

Authors: Helge Aasen, Norbert Kirchgessner, Achim Walter, Frank Liebisch

**Fig. S1** Some plots already start to yellow at DAS 99 (image on the left, brightness adjusted for visualization purposes) and continue very slow (right)


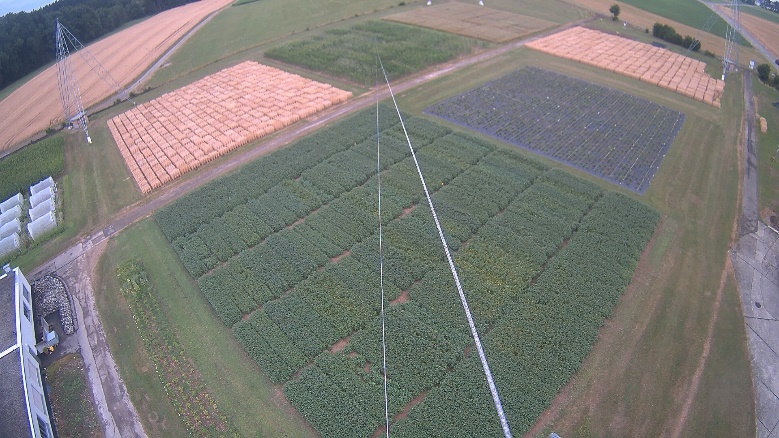

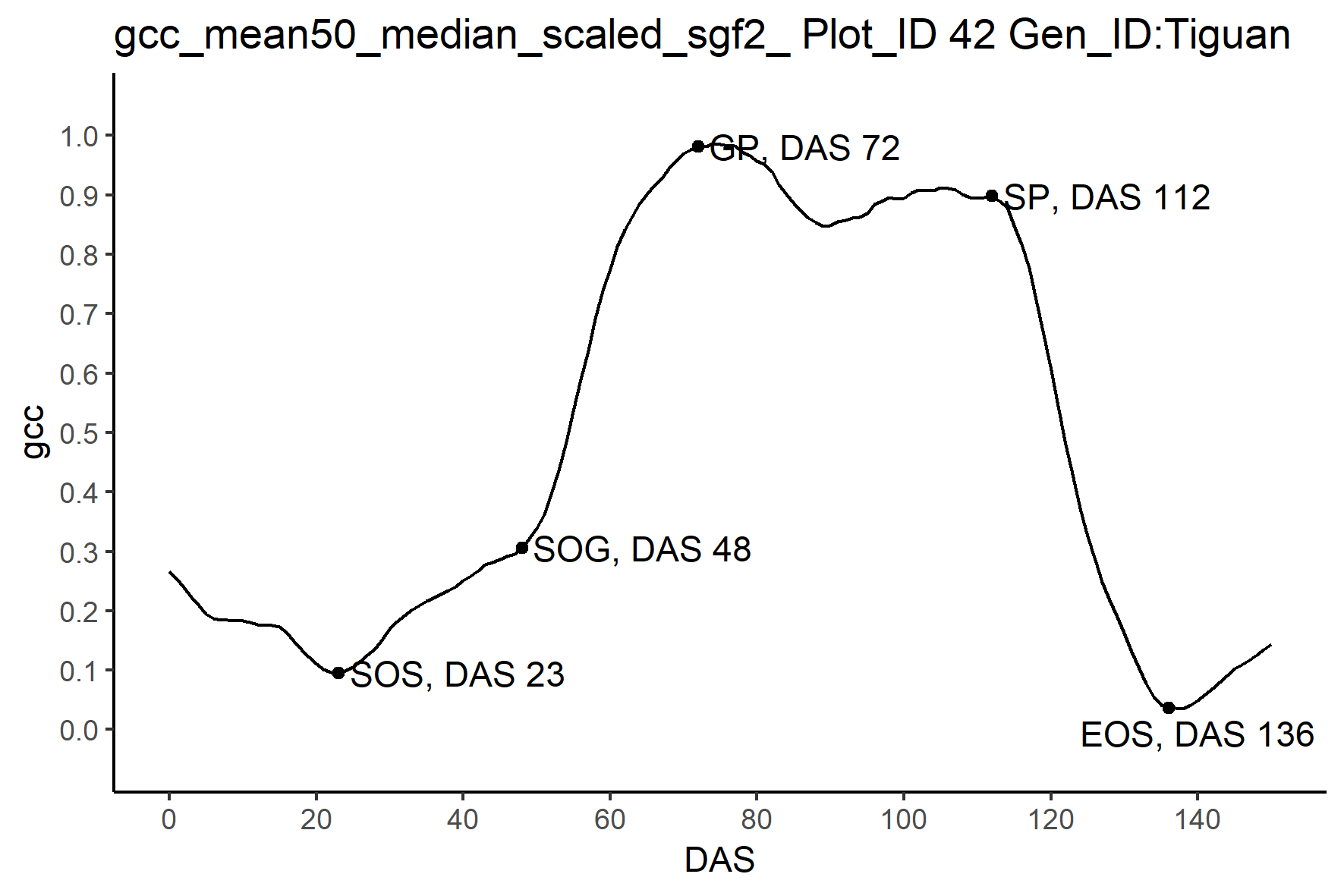


**Table S1** Summary statistics of the PhenoTimePoint and PhenoPhase timings in days after sowing (DAS) and growing degree days (GDD) along with their heritability (H2).

|  | **mean** | | **min** | | **max** | | **range** | |  |
| --- | --- | --- | --- | --- | --- | --- | --- | --- | --- |
|  | **DAS** | **GDD** | **DAS** | **GDD** | **DAS** | **GDD** | **DAS** | **GDD** | **H2** |
| SOG | 48 | 352 | 47 | 337 | 50 | 382 | 3 | 45 | 0.85 |
| GP | 76 | 729 | 72 | 679 | 79 | 778 | 7 | 99 | 0.76 |
| SP | 125 | 1553 | 116 | 1400 | 131 | 1660 | 15 | 260 | 0.97 |
| EOS | 144 | 1828 | 139 | 1749 | 147 | 1874 | 8 | 125 | 0.88 |
| EOS_SP | 22 | 303 | 19 | 238 | 26 | 378 | 8 | 140 | 0.68 |
| EOS_GP | 68 | 1112 | 65 | 1074 | 72 | 1161 | 7 | 87 | 0.46 |
| EOS_SOG | 97 | 1489 | 92 | 1417 | 101 | 1548 | 9 | 131 | 0.86 |
| SP_GP | 49 | 828 | 45 | 727 | 55 | 921 | 10 | 194 | 0.89 |
| SP_SOG | 76 | 1194 | 68 | 1047 | 82 | 1295 | 14 | 248 | 0.96 |
| GP_SOG | 28 | 383 | 25 | 340 | 30 | 407 | 6 | 67 | 0.68 |

**Table S2** Cultivation measures classified into categories and treatments. Products and their amount per lot are given as well as the application date.

| **Category** | **Treatment** | **Product (amount/lot)** | **Date** |
| --- | --- | --- | --- |
| **Soil cultivation** | plow |  | 24.11.2014 |
| **Fertilization** | fertilizer application | Superphosphat (22.68 kg) | 11.03.2015 |
| **Fertilization** | fertilizer application | Foskal (52.92 kg/lot) |  |
| **Fertilization** | fertilizer application | Dolomit 55% CaCo3, 35% Mg CO3 (60.48 kg) |  |
| **Plant protection** | herbicide application | Dual Gold (0.3 l) | 10.04.2015 |
| **Plant protection** | molluscicide application | slug pellets PS (1.06 kg) |  |
| **Plant protection** | herbicide application | Molipan Pro (0.15 kg) | 14.04.2015 |
| **Plant protection** | herbicide application | Bolero (0.15 l) | 28.05.2015 |
| **Plant protection** | herbicide application | Fusilade Max (0.45 l) | 10.06.2015 |
| **Harvest** |  |  | 10.09.2015 |

**Table S3** Coefficient of determination of the relationship between the zenith angle and the phenotypic (blue) and spatially corrected genotypic (red) values of the (a-f) phenological signal (gcc) at different days after sowing (DAS) and for different PhenoTimePoints (g-j). This table corresponds to Figure 5 of the publication.

| **DAS** | **phenotypic** | **(p-value)** |  | **genotypic** | **(p-value)** |
| --- | --- | --- | --- | --- | --- |
| 40 | 0.07 | 0.02 |  | 0.00 | 0.54 |
| 50 | 0.08 | 0.01 |  | 0.00 | 0.55 |
| 61 | 0.00 | 0.79 |  | 0.00 | 0.74 |
| 80 | 0.55 | 0.00 |  | 0.00 | 0.61 |
| 120 | 0.26 | 0.00 |  | 0.00 | 0.86 |
| 142 | 0.04 | 0.06 |  | 0.00 | 0.69 |
| SOG | 0.01 | 0.00 |  | 0.04 | 0.00 |
| GP | 0.09 | 0.00 |  | 0.02 | 0.00 |
| SP | 0.06 | 0.00 |  | 0.00 | 0.11 |
| EOS | 0.02 | 0.00 |  | 0.00 | 0.15 |
